# Spatial distribution and drivers of arbuscular mycorrhizal fungi on the Tibetan Plateau

**DOI:** 10.3389/fpls.2024.1427850

**Published:** 2024-06-20

**Authors:** Feng Zhang, Yaoming Li, Baoming Ji, Shikui Dong

**Affiliations:** School of Grassland Science, Beijing Forestry University, Beijing, China

**Keywords:** arbuscular mycorrhizal fungi, biodiversity, alpine grassland, community assembly mechanism, Tibetan Plateau

## Abstract

**Introduction:**

Arbuscular mycorrhizal fungi (AMF) are pivotal in plant resource acquisition, mediating plant interactions, and influencing soil carbon dynamics. However, their biogeographical distribution in Tibetan alpine grasslands remains understudied.

**Methods:**

In this research, we examined the distribution pattern of AMF communities and their key determinants along a 2000-km transect across the Tibetan plateau, encompassing 7 alpine meadows and 8 alpine steppes.

**Results:**

Our findings indicate that AMF community diversity and composition exhibit similarities between alpine meadows and alpine steppes, primarily influenced by latitude and evapotranspiration. At the genus level, *Glomus* predominated in both alpine meadow (36.49%±2.67%) and alpine steppe (41.87%±2.36%) soils, followed by *Paraglomus* (27.14%±3.69%, 32.34%±3.28%). Furthermore, a significant decay relationship of AMF community was observed over geographical distance. Null model analyses revealed that random processes predominantly (>50%) drove the assembly of AMF communities.

**Discussion:**

In summary, our study elucidates the spatial distribution pattern of AMF in Tibetan plateau grasslands and underscores the significant influence of geographical and climatic factors on AMF community dynamics.

## 1 Introduction

Microorganisms play a crucial role in ecosystem functions, with soil serving as an abundant microbial reservoir for host plants (Sokol et al., 2022). While ecologists have extensively studied the biotic and abiotic processes influencing above-ground organisms (Mod et al., 2020; Liu et al., 2021; Xi et al., 2023), below-ground microbial communities have received less attention (Ott et al., 2019; Chu et al., 2020).

Arbuscular mycorrhizal fungi (AMF) are widely distributed soil microorganisms capable of forming beneficial associations with over 80% of vascular plants (Dietrich et al., 2020; Giovannini et al., 2020; Gujre et al., 2021). These fungi contribute to plant nutrient acquisition and play pivotal roles in ecosystem functioning and sustainability (Berdeni, 2016; Li et al., 2020). Understanding the relationship between the diversity and distribution of AMF taxa and environmental conditions across spatial gradients is essential to elucidate their impact on ecosystem functions (Meyer et al., 2018). Various factors influence the spatial patterns of AMF communities in grassland soils, including climate (Zhang et al., 2016; Wang et al., 2021), geochemical properties (You et al., 2023), and soil type (Oehl et al., 2010). However, consistent conclusions regarding AMF biogeographic patterns and their controlling factors have yet to be reached (Xiang et al., 2014; Davison et al., 2015). Some studies emphasize edaphic factors and climate parameters in explaining the variance in AMF distribution patterns (Ma et al., 2022; Ma et al., 2023a; Ma et al., 2023b)—for instance, soil texture and fertility are identified as key drivers determining the AMF community structure (Goldmann et al., 2020; Aker et al., 2022; Zhang et al., 2022; Liu et al., 2023).

The Tibetan Plateau, with an average altitude exceeding 4,000 m, is the primary distribution area for grasslands in China. Additionally, as the world’s highest and most distinctive regional unit, boasting a fragile ecological environment, it serves as an indicator and regulator of global climate and environmental changes, representing a crucial ecological security barrier in China (Yao et al., 2012; Feng et al., 2021). Alpine grassland is the predominant ecological type on the Tibetan Plateau. Due to the limitations of extreme environments such as low temperatures, drought, and barrenness, AMF have evolved many coping strategies to survive in the long-term evolutionary process. However, differences across AMF communities originating from different climatic zones have been detected, underscoring the importance of climate factors (Islam et al., 2020; Paranavithana et al., 2021). Geographic constraints, particularly geographical distance (Sanguin et al., 2016; Valentin et al., 2023) and mean annual precipitation (MAP) (Zhang et al., 2016; Wang et al., 2021), have also been proposed as significant drivers of AMF distribution. Thus, our current understanding of the geographic distributions of AMF species and the underlying mechanisms remains limited, especially in alpine ecosystems.

In this study, we elucidate the distribution pattern of AMF communities and the corresponding drivers in surface soils (0–10 cm) collected from 90 grassland samples on the Tibetan Plateau spanning over 2,000 km. These grasslands exhibit high heterogeneity in plant community composition and abiotic factors. Our objective is to investigate the following: (1) the spatial distribution pattern of AMF species in Tibetan Plateau grasslands at a regional scale and (2) the dominant environmental drivers controlling AMF community composition.

## 2 Materials and methods

### 2.1 Study sites and soil sampling

**Figure 1:**
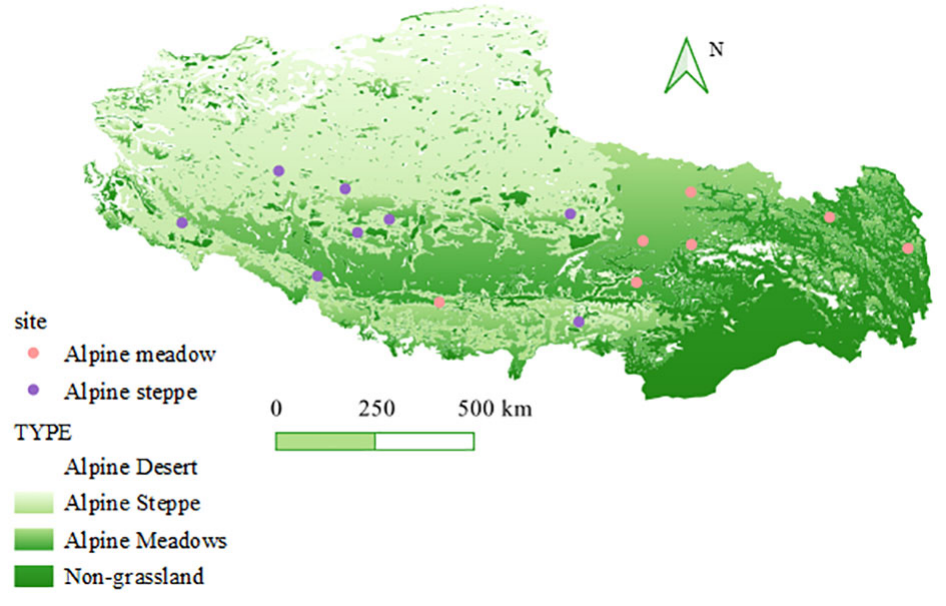
Site location and grassland type of the 15 sampling sites for arbuscular mycorrhizal fungi survey in the Tibetan Plateau.

Rhizosphere soils were collected from 15 alpine grasslands (including 48 alpine steppes and 42 alpine meadows) along a 3,849-m to 5,299-m elevational gradient (23.43°–28.76° N, 80.86°–98.51° E) on the Tibetan Plateau (**Figure 1**; **Supplementary Table S1**) in August, 2021. Peak vegetation growth and maximum microbial activity and biomass were found in this season. The selected sites in this conducted survey include both typical grassland types here, i.e., alpine meadow and alpine steppe. In addition, the sites are characterized by considerable variations in mean annual temperature (MAT) (from -4.35°C to 6.55°C) and mean annual precipitation (MAP) (from 3,849 to 5 299 m; 1980–2015). These data were obtained from the Institute of Geographic Sciences and Natural Resources Research, CAS (https://www.resdc.cn/Default.aspx).

At each of the 15 grassland sites, six quadrats (1 m × 1 m) were laid out in a Nestle-style pattern, totaling 90 quadrats (6 × 15) (**Figure 1**). Subsequently, five soil cores (of 5-cm diameter and 10-cm depth) were collected in each quadrat, thoroughly homogenized to form a composite sample, and sieved to no more than 2 mm. Then, the soils at field moisture were divided into two subsamples (200 g each). The soils were kept on ice in the field and shipped overnight on dry ice to Beijing Forestry University. One subsample was air-dried for the determination of physicochemical soil properties, while the other was kept at −80°C in the laboratory for DNA extraction, PCR amplification, and sequencing.

### 2.2 Soil physicochemical determination and plant data

Soil physiochemical properties were measured as previously described (Lu, 2000). The soil moisture was measured by putting 5 g of soil into an oven at 105°C until a constant weight was reached. Soil pH and electro-conductibility (EC) were measured in a soil suspension with a soil/water ratio of 1:2.5 (weight/volume) using a combo pH and EC (HI, 98130, Hanna Instrument). The soil C and N contents were measured by using LECO TruSpec Carbon and Nitrogen Analyzer (LECO Corporation, St. Joseph, MI). Soil NH_4_^+^ and NO_3_^−^ contents were extracted from the soils with 1 M KCl and measured by using Lachat QuikChem, 8500 series 2 instrument (Lachat, Loveland, CO, USA). The Normalized Difference Vegetation Index (NDVI) at each site around the sampling time was collected from Moderate Resolution Imaging Spectroradiometer (MODIS).

### 2.3 DNA extraction, PCR, and amplicon sequencing

Soil DNA was extracted from 0.5 g of soil using a FastDNA spin kit for soil (Tiangen, China) following the manufacturer’s instructions. The AMF-specific primer pair AMV4.5NF (5′-AAGCTCGTAGTTGAATTTCG-3′) and AMDGR (5′-CCCAACTATCCCTATTAATCAT-3′) was used to amplify 18S rRNA gene, and PCR reaction conditions were adapted from a previous report (Suzuki et al., 2020). The PCR products were pooled at equal molality and sequenced on an Illumina Hiseq, 4000 platform (Meige Company, Shenzhen, China).

### 2.4 Sequence and bioinformatic analysis

The sequences were trimmed, quality-filtered, and de-replicated, and amplicon sequence variant (ASV) tables were generated using the DADA2 pipeline in QIIME II, according to previous reports (Blaalid and Khomich, 2021). The reads were truncated at 225 bp, corresponding to a quality score of >20. For taxa comparisons, we used the QIIME2 q2-feature-classifier plugin and the naïve Bayes classifier that was trained on the MaarjAM using the Scikit-learn feature classifier (Opik et al., 2010), revealing 89 virtual taxa (VT; approximately species-level phylogroups). The samples were retained if they contained >100 reads, while global singleton VT were omitted, leaving 89 VT in 90 samples. The VT count tables were further sub-sampled (rarefied) to even depths of 5,000 sequences before computing the alpha- and beta-diversities (in QIIME2) and following the statistical analyses using R. Paired-end sequence reads generated from this study have been deposited in the sequence read archives of the National Center for Biotechnological Information under BioProject ID PRJNA1104783.

### 2.5 Data analyses

Data analysis was conducted by using the packages vegan (Dixon, 2003), plspm (Dijkstra and Henseler, 2015), picante (Kembel et al., 2010), and icamp (Ning et al., 2020) with the statistical platform R version 4.3.0. Bacterial beta-diversity was estimated as the average pairwise community dissimilarity within each sample using Bray–Curtis distance matrices by permutation multivariate dispersion (PERMDISP). Mantel tests were performed to evaluate the influence of environmental and geographic variables on AMF community composition (soil nutrient content, climate elements, and plant elements) (Guillot and Rousset, 2013). We then used the package plspm to better understand the causal relationship of each environmental variable influence, with a structural equation model (SEM) constructed. Non-metric multidimensional scaling (NMDS) analysis was used to visualize the sample relationships across different sites in overall community composition (Oksanen et al., 2012). A total of 18 plant and soil variables were performed to evaluate possible linkages between bacteria and these variables.

In order to explore the community construction mechanism of AMF, community Construction Analysis Framework based on Stegen-QPEN (Quantifying assembly Processes based on Entire-Community Null model analysis) was applied (Stegen et al., 2012, 2013). First, the degree of phylogenetic turnover (βMNTDobs) in each paired community was quantified and compared with the null distribution (βMNTDnull). The βNTI value represented the size of the deviation between βMNTDobs and βMNTDnull. Significant βNTI values (|βNTI| >2) were the result of choice, and βNTI <-2 and βNTI >2 was the choice of homogeneous and heterogeneous, respectively. Not significant for βNTI values (|βNTI| <2), based on the Bray–Curtis Raup–Crick (RC Bray) value representation BCobs deviation between BCnull and size, usually RCbray <-0.95 is defined as homogeneous diffusion, RCbray >0.95 represents the diffusion limit, and |RCbray| <0.95 is on behalf of the drift. Detailed descriptions could be found in previous studies (Stegen et al., 2012; Dini-Andreote et al., 2015). The statistical significance of those comparisons was determined using 999 permutations, and the analyses were carried out using the icamp package for R.

## 3 Results

### 3.1 Soil and climate properties

**Figure 2:**
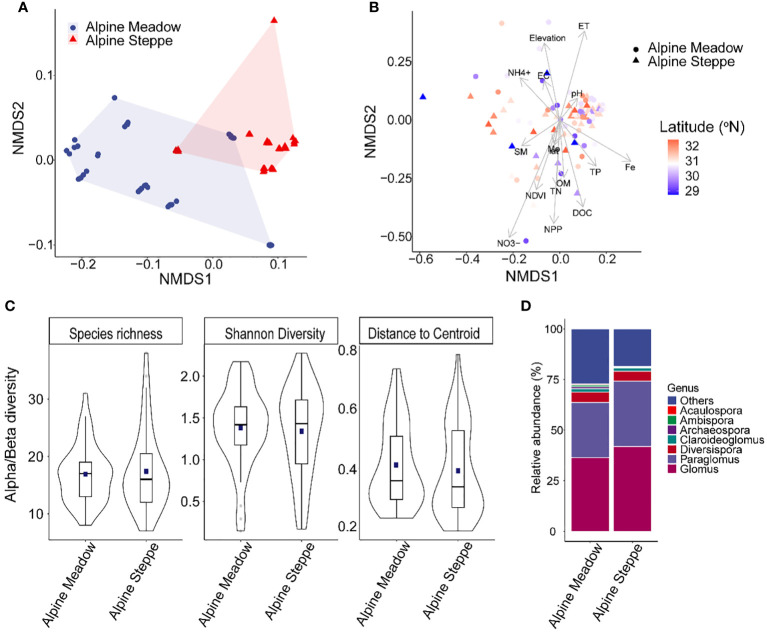
Non-metric multidimensional scaling ordination plots of dissimilarities for environmental properties **(A)** and arbuscular mycorrhizal fungi (AMF) virtual taxa (VT) composition **(B)** of alpine meadows and alpine steppes. Species (i.e., VT) richness, Shannon diversity, and beta diversity (measured and distance to centroid) **(C)** and genus composition **(D)** of AMF community of alpine meadows and alpine steppes.

Soil and climate properties were significantly different between alpine meadow and steppe (**Figure 2A**). Alpine meadow showed significantly higher values in soil nutrient content, MAT, MAP, and NDVI, but lower pH values, compared with alpine steppe soils (**Supplementary Figure S1**).

### 3.2 AMF community properties

**Figure 3:**
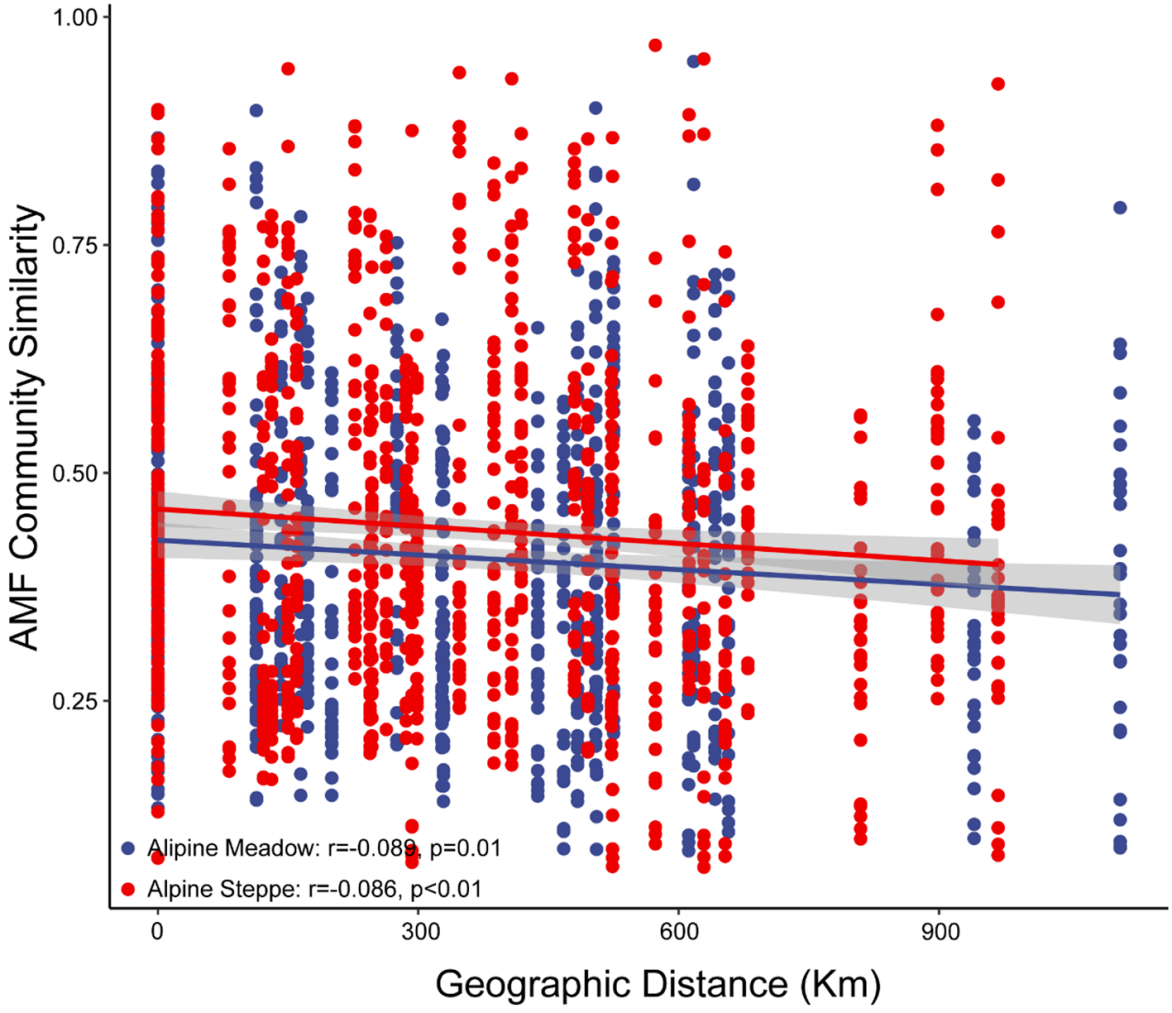
Distance–decay relationships of arbuscular mycorrhizal fungi communities (based on Bray–Curtis distances) in alpine meadow and steppe.

The AMF virtual taxa (VT) composition and diversity were similar between the two alpine grassland types. Furthermore, 89 AMF virtual taxa (VT) representing 10 genera were detected across the two alpine grassland types (**Figure 2D**). VT richness was not significantly different between alpine meadow (16.9 ± 0.79) and steppe (17.4 ± 0.17%), neither for Shannon diversity (**Figure 2C**). Similarly, beta diversity (measured as the distance to group centroid, *F* = 0.4, *p* = 0.52) (**Figure 2C**) and AMF VT composition were not significantly different between the two grassland types (ADONIS, *R* ^2 ^=^ ^0.01, *p* = 0.54) (**Figure 2B**). *Glomus* was most abundant in both alpine meadow (36.49% ± 2.67%) and alpine steppe (41.87% ± 2.36%) soils, followed by *Paraglomus* (27.14% ± 3.69% in alpine meadow and 32.34% ± 3.28% in alpine steppe) (**Figure 2D**). The AMF community similarity in alpine meadow (*r* = -0.089, *p* = 0.01) and steppe (*r* = -0.086, *p* < 0.01) significantly decreased over geographic distance (**Figure 3**).

### 3.3 Relationship between AMF community composition and environmental variables

The major abiotic factors influencing AMF community composition were identified using Mantel test. The structure of soil AMF community was significantly influenced by latitude (*r* = 0.13, *p* = 0.01) and ET (*r* = 0.13, *p* = 0.02), but not the other 20 soil and climate variables (**Table 1**). These variables were further retained by the forward selection process and used in canonical correspondence analysis models, respectively. Soil properties explained only 5.9% of the variation compared with 12% by climate conditions of the AMF community.

**Table 1:** Mantel tests between environmental variables and the arbuscular mycorrhizal fungi community composition using Pearson and Spearman correlation.

	*R*	*P*	Method	*R*	*p*	Method
Latitude	0.13	0.01	Pearson	0.10	0.032	Spearman
Elevation	0.07	0.138	Pearson	0.06	0.145	Spearman
NDVI	0.05	0.168	Pearson	0.04	0.18	Spearman
ET	0.13	0.016	Pearson	0.09	0.0127	Spearman
NPP	0.05	0.126	Pearson	0.07	0.061	Spearman
OM	0.06	0.134	Pearson	0.06	0.148	Spearman
TN	0.04	0.242	Pearson	0.06	0.154	Spearman
TP	0.09	0.073	Pearson	0.07	0.13	Spearman
SM	-0.02	0.646	Pearson	-0.02	0.634	Spearman
DOC	0.08	0.165	Pearson	0.04	0.253	Spearman
NH_4_^+^–N	-0.02	0.551	Pearson	-0.04	0.713	Spearman
NO_3_^–^N	0.01	0.423	Pearson	0.03	0.319	Spearman
pH	0.07	0.06	Pearson	0.06	0.07	Spearman
EC	-0.01	0.467	Pearson	0.04	0.284	Spearman
Fe	0.01	0.34	Pearson	0.02	0.259	Spearman
Mo	-0.02	0.613	Pearson	-0.03	0.684	Spearman

NDVI, normalized difference vegetation index; ET, evapotranspiration; NPP, net primary production; OM, soil organic matter; TN, total nitrogen; TP, total phosphorus; SM, soil moisture; DOC, dissolved organic carbon; NH_4_^+^–N, soil nitrate N contents; NO_3_^—^N, soil ammonium N contents; EC, electroconductibility.

**Figure 4:**
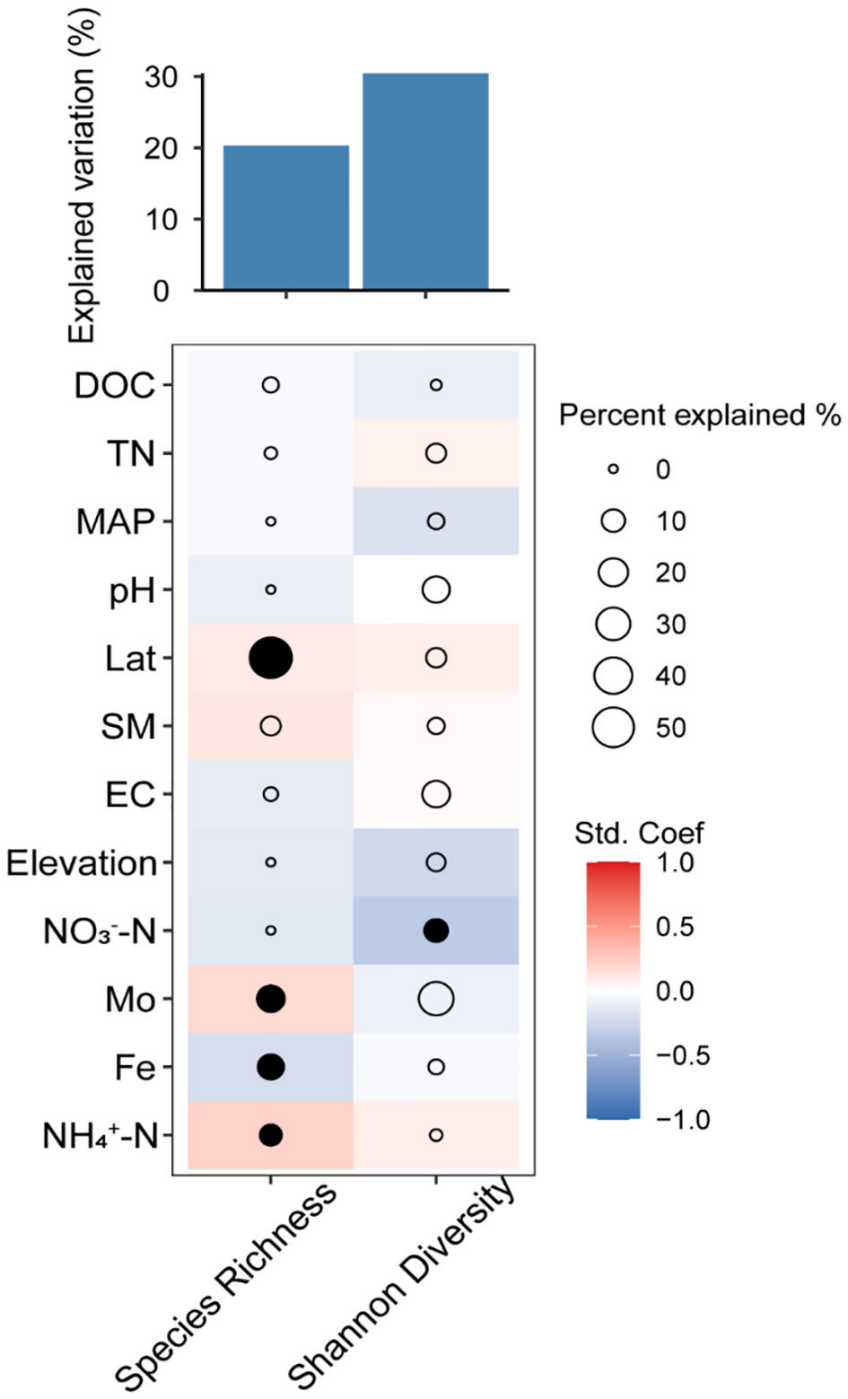
Percentage of the total variance in arbuscular mycorrhizal fungi (AMF) α-diversity explained by environmental factors and the correlations between them. DOC, dissolved organic carbon; TN, total nitrogen; MAP, mean annual precipitation; Lat, latitude; SM, soil moisture; EC, electroconductibility.

**Figure 5:**
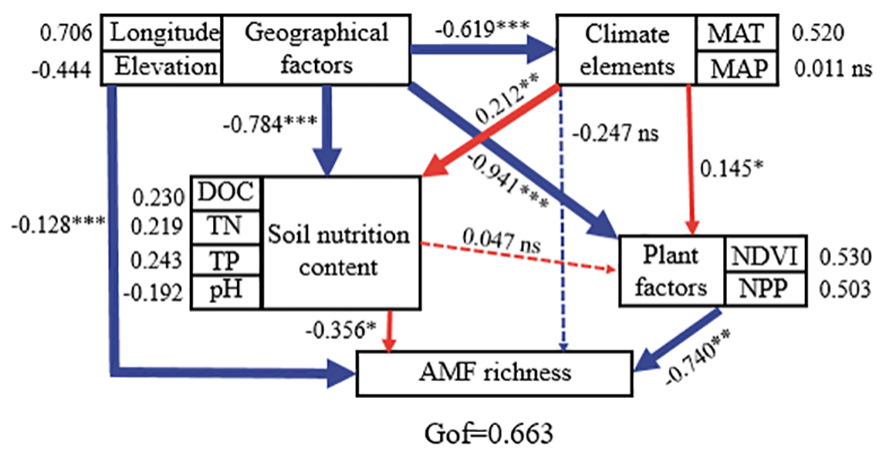
Structure equation model to quantify the effects of geographic factors, climate elements, soil nutrition, and plant factors on soil arbuscular mycorrhizal fungi community in Tibetan alpine grasslands. The red and blue lines stand for positive and negative correlations, respectively; the bold lines stand for significance at *p* = 0.05 level. ns indicates that there is no significant difference between treatments.

Similarly, latitude showed the greatest effect size on AMF species richness, followed by Mo, Fe, and NH_4_^+^–N concentrations (**Figure 4**). Additionally, latitude, Mo, and NH_4_^+^–N concentrations have a positive effect on AMF species richness, while Fe concentration showed a negative effect. Furthermore, we combined structural equation model (SEM) analysis to further quantify the contributions of the measured factors to AMF diversity. As suggested by the SEM analysis, geographical factors (longitude and elevation) had strong influences on soil and climate properties which strongly impact the AMF community (**Figure 5**). All measured variables explained 20.31% of the variation in AMF species richness (**Figure 4**). Soil NO_3_^–^N had a negative effect on AMF Shannon diversity, and the measured variables explained 30.41% of the variation in AMF Shannon diversity (**Figure 4**).

### 3.4 Relative effects of ecological processes on community assembly

We estimate the community assembly processes using Null model and βNTI (β-nearest taxon index) analyses. Due to the similar AMF community properties and distribution patterns in alpine meadow and steppe soils, we merged all the samples to conduct the analysis. Null model analysis showed that neutral assembly, including dispersal limitation (88.64%) and drift (4.84%), was dominant in the AMF community in the soil. Dispersal limitation accounted for 88.64%.

The relationships between βNTI and major factors were used to infer changes in the relative influences of deterministic and stochastic assembly processes. We chose AMF richness to be the predictor of βNTI. The degree of phylogenetic turnover in each paired community was quantified (βMNTDobs) and compared with the zero distribution (βMNTDnull). The βNTI value represented the size of the deviation between βMNTDobs and βMNTDnull. With the increase of AMF species richness, the relative influence of random community construction process decreased significantly. There was no significant correlation between RC and the AMF species richness.

## 4 Discussion

We investigated the distribution of AMF communities and their key predictors across a 2,000-km transect on the Tibetan plateau, including alpine meadow and alpine steppe. The non-significant differences in AMF composition and diversity between the alpine grasslands indicate less influences of vegetation type compared with soil and climate factors. Although there is accumulating knowledge about how AMF assemblages interact with plants, the nature and direction of these interrelations are not entirely clear. Some results found a significant relationship between AMF distribution patterns and occurrence in plant host, suggesting a spatial co-variation of vegetation and of AMF communities in landscapes (Oepik et al., 2009). Two decades ago, Hart and Klironomos presented the driver and passenger hypotheses to describe two general ways in which AMF and plant communities might change over space (Hart and Klironomos, 2001). According to the passenger hypothesis, the AMF community distribution pattern is controlled by changes in plant communities. However, Zobel and Oepik introduced the habitat hypothesis: the dynamics of AMF are not related to plant communities but the soil and climate conditions (Zobel and Oepik, 2014). Our results followed the habitat hypothesis. Latitude was the most important factor in impacting AMF community composition and species richness, through affecting soil and climate conditions.

Our result showed that *Glomus* and *Paraglomus* were the most abundant arbuscular mycorrhizal fungi genus in the Tibetan Plateau grassland soils, which were consistent with the results of previous studies (Malar et al., 2022; Velez-Martinez et al., 2023; Li et al., 2024). These species may be more adaptable in adjusting the patterns of sporulation to environmental stress conditions of the alpine grasslands, as evidenced by its global distribution (INVAM biogeographical database; https://www.invam.caf.wvu.edu). Different AM fungal groups have different effects on plant performance and nutrient cycling. Studies have documented the effectiveness of *Glomus* spp. to improve the performance of the target plant species. Additionally, *Paraglomus* has been associated with soils rich in plant diversity and forested areas (Marinho et al., 2018; Pena-Venegas et al., 2021).

In this study, we observed that across all abiotic (e.g., geographical and climatic) factors, ET strongly correlated with AMF community composition, which was supported by a previous study (Zhang et al., 2018; Jerbi et al., 2022). This highlights the vital role of water availability in driving the distribution of AMF microorganism in Tibetan soils. First, water is widely recognized as a main limiting factor for soil microbes and plays a key role in structuring soil microbial communities (Allsup et al., 2022; Liang et al., 2022; Guo et al., 2024). Hence, it is persuasive to assert that water availability may directly drive the AMF distribution on the Tibetan Plateau. Second, water availability is also a determinative factor for plant growth and community composition (Lin et al., 2021; Joachin et al., 2023; Lin et al., 2023), affecting the availability and quality of organic carbon and light, and thus indirectly drives the distribution of soil AMF. Collectively, water content might influence the distribution of Tibetan soil AMF in both direct and indirect manners, which is also supported by the pathway analysis in previous studies (Wilson et al., 2016). While AMF played a role in the absorption of phosphorus (P) and nitrogen (N) in plants, there was no significant correlation between P and N and AMF composition in this study. On one hand, the study area was located on the Tibetan Plateau, where altitude significantly influenced the composition of AMF communities (Li et al., 2018). The high altitude of this study may have contributed to the differences in the results. On the other hand, in alpine regions, AMF not only assists plants in nutrient absorption but also helps plants cope with environmental stress. As Chen et al. (2013) have suggested, although plants reduced their dependence on AMF for nutrient absorption under nutrient-sufficient conditions, they still required AMF to help them resist environmental stress. Therefore, the combined effects of these two factors contribute to the insignificant impact of nitrogen and phosphorus on the composition of AMF communities.

It is of great significance to reveal the assembly mechanism of underground microbial community from the perspective of microbial ecology (Nemergut et al., 2013), which had been explored extensively (Zhou and Ning, 2017), yet it is widely acknowledged that both deterministic and stochastic processes influence the biogeographic patterns of microbial communities and distance–decay relationships. (i.e., microbial community similarity decreases as geographical distance increases) (Wang et al., 2017). Deterministic processes involve nonrandom and niche-based mechanisms (Vellend, 2010), including environmental filtering and interspecific interactions. In contrast, stochastic processes mainly reflect random changes in the relative abundance of species, involving random birth, death, and dispersal events (Hubbell, 2001; Chase and Myers, 2011). Species interactions, which could determine the functional attributes or niche occupancy of microbial communities (Freilich et al., 2010; Becker et al., 2012), play important roles in stimulating ecosystem processes (Bardgett et al., 2014).

## 5 Conclusion

The alpine grassland ecosystem, serving as a pivotal research focus in the context of global climate change, is also a crucial ecological barrier in China. It holds a significant role in sustaining the ecological balance, particularly in arid, alpine, and other environmentally disadvantaged regions, thus possessing exceptional ecological importance. Our study showed that the AMF microorganisms on the Tibetan Plateau grassland soils were dominated by *Glomus* and *Paraglomus.* The distribution patterns of soil AMF diversity and community composition on the Tibetan Plateau alpine grasslands are strongly affected by latitude and ET. There was a significant attenuation relationship between community composition and geographical distance. Moreover, the random assembly was dominant (>50%) in AMF community assembly. This was an important finding in the understanding of alpine ecosystems, predicting their responses to climate change, and informing effective conservation and management strategies. Further studies are now needed to elucidate linkages between AMF function and biogeography. However, in this study, the AMF microorganisms were analyzed using DNA-based methods which failed to identify the AMF’s activity.

## Data availability statement

The datasets presented in this study can be found in online repositories. The names of the repository/repositories and accession number(s) can be found below: https://www.ncbi.nlm.nih.gov/genbank/, PRJNA1104783.

## Author contributions

FZ: Writing – original draft, Writing – review & editing. YL: Conceptualization, Data curation, Investigation, Methodology, Writing – original draft, Writing – review & editing. BJ: Writing – review & editing. SD: Conceptualization, Writing – review & editing.
