# Supplementary material for: Spatial distribution and drivers of arbuscular mycorrhizal fungi on the Tibetan Plateau
Source: Front Plant Sci. 2024 Jun 20;15:1427850. doi: 10.3389/fpls.2024.1427850 (PMC11264307; doi:10.3389/fpls.2024.1427850)
Supplement: Supplementary file 1 [file DataSheet_1.docx]

**ZHANG et al. “Spatial distribution and drivers of arbuscular mycorrhizal fungi on the Tibetan Plateau”** **supplementary materials**

Table S1. Sampling information for the research sites. MAT: Mean Annual Temperature. MAP: Mean Annual Precipitation

| Site | Location | Grassland type | longitude | longitude | Elevation | MAT | MAP |
| --- | --- | --- | --- | --- | --- | --- | --- |
| S1 | Gaer | Alpine steppe | 80.86 | 31.17 | 4634 | 0.07 | 378.07 |
| S2 | Gaize | Alpine steppe | 83.21 | 32.43 | 4442 | -0.23 | 212.51 |
| S3 | Zhongba | Alpine steppe | 84.16 | 29.88 | 4673 | 0.52 | 518.16 |
| S4 | Dongcuo | Alpine steppe | 84.83 | 31.99 | 4586 | -0.48 | 267.20 |
| S5 | Cuoqin | Alpine steppe | 85.13 | 30.93 | 4693 | 0.38 | 345.20 |
| S6 | Nima | Alpine steppe | 85.90 | 31.25 | 4752 | -0.07 | 325.72 |
| S7 | Angren | Alpine meadow | 87.12 | 29.23 | 4835 | 2.49 | 446.65 |
| S8 | Bange | Alpine steppe | 90.30 | 31.38 | 4629 | 0.11 | 432.67 |
| S9 | Langkazi | Alpine steppe | 90.51 | 28.76 | 4471 | 2.55 | 487.75 |
| S10 | Mozugongka | Alpine meadow | 91.91 | 29.72 | 4087 | 4.13 | 622.15 |
| S11 | Anduo | Alpine meadow | 92.07 | 30.73 | 5299 | -4.35 | 737.52 |
| S12 | Naqu | Alpine meadow | 93.23 | 31.92 | 4615 | -0.20 | 620.52 |
| S13 | Jiali | Alpine meadow | 93.25 | 30.64 | 4504 | 1.33 | 721.30 |
| S14 | Leiwuqi | Alpine meadow | 96.61 | 31.30 | 3849 | 4.42 | 603.18 |
| S15 | Gongjue | Alpine meadow | 98.51 | 30.55 | 3923 | 6.55 | 644.55 |


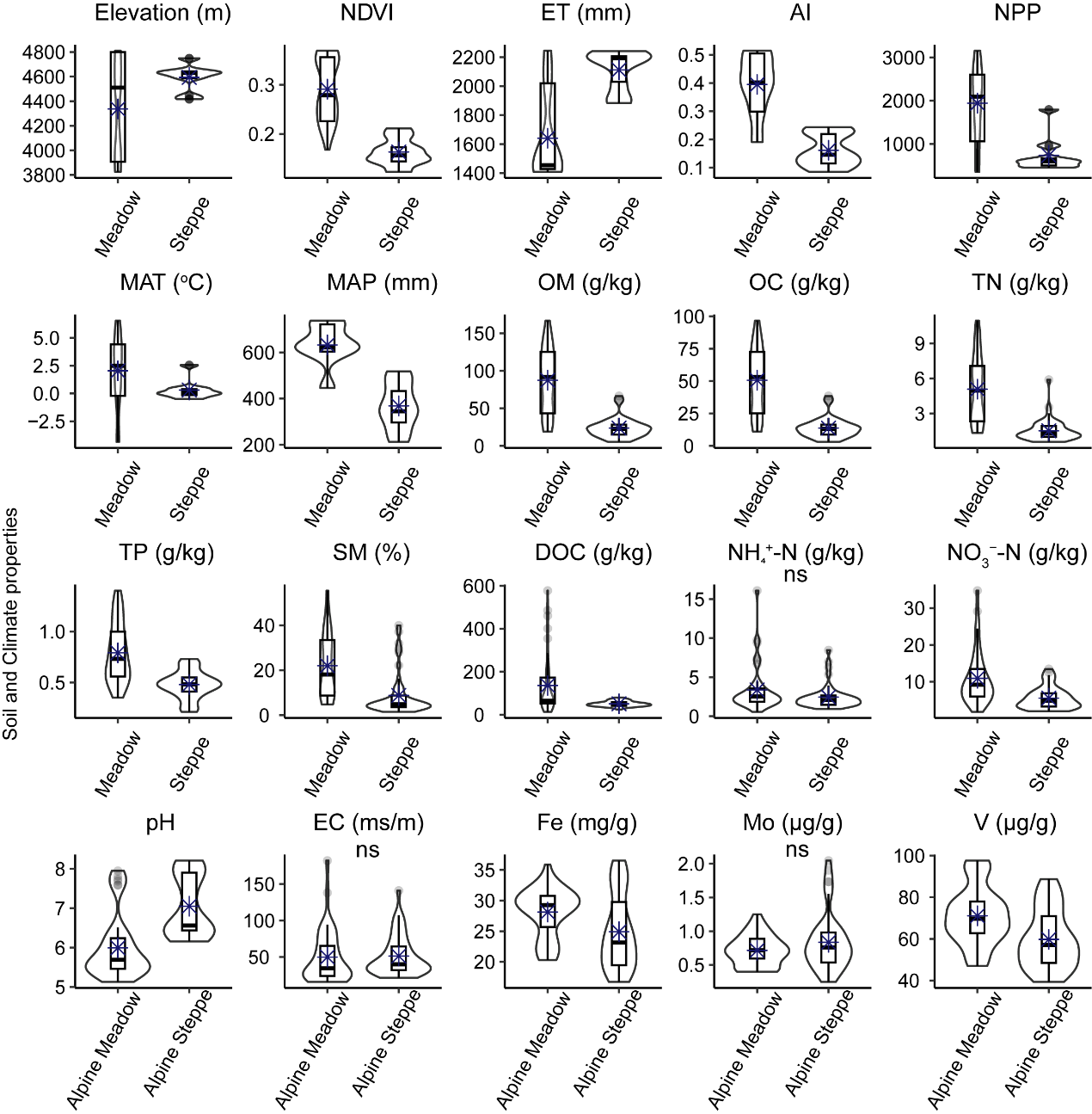


Figure S1. Environmental variables of alpine meadows and alpine steppes. NDVI: normalized difference vegetation index. ET: evapo Transpiration. NPP: net primary production. OM: soil organic matter. OC: soil organic carbon. TN: total nitrogen. TP: total phosphorus. SM: soil moisture. DOC: dissolved organic carbon. NH_4_^+^-N: soil nitrate N contents. NO_3_^-^-N: soil ammonium N contents. EC: electroconductibility. MAT: mean annual temperature. MAP: mean annual precipitation.
